# The association between lymphocyte-to-monocyte ratio and all-cause mortality in obese hypertensive patients with diabetes and without diabetes: results from the cohort study of NHANES 2001–2018

**DOI:** 10.3389/fendo.2024.1387272

**Published:** 2024-04-15

**Authors:** Lixia Wang, Jie Gao, Bing Liu, Youliang Fu, Zhihui Yao, Shanshan Guo, Ziwei Song, Zhaoyuan Zhang, Jiaojiao He, Congxia Wang, Weidong Ma, Feng Wu

**Affiliations:** ^1^ Department of Cardiology, Xi’an International Medical Center Hospital, Xi’an, Shaanxi, China; ^2^ Department of Cardiology, Second Affiliated Hospital of Xi’an Jiaotong University, Xi’an, Shaanxi, China

**Keywords:** lymphocyte-to-monocyte ratio (LMR), all-cause mortality, obesity, hypertension, diabetes

## Abstract

**Objective:**

Obesity, hypertension and diabetes are high prevalent that are often associated with poor outcomes. They have become major global health concern. Little research has been done on the impact of lymphocyte-to-monocyte ratio (LMR) on outcomes in these patients. Thus, we aimed to explore the association between LMR and all-cause mortality in obese hypertensive patients with diabetes and without diabetes.

**Methods:**

The researchers analyzed data from the National Health and Nutrition Examination Survey (2001-2018), which included 4,706 participants. Kaplan-Meier analysis was employed to compare survival rate between different groups. Multivariate Cox proportional hazards regression models with trend tests and restricted cubic splines (RCS) analysis and were used to investigate the relationship between the LMR and all-cause mortality. Subgroup analysis was performed to assess whether there was an interaction between the variables.

**Results:**

The study included a total of 4706 participants with obese hypertension (48.78% male), of whom 960 cases (20.40%) died during follow-up (median follow-up of 90 months). Kaplan–Meier curves suggested a remarkable decrease in all-cause mortality with increasing LMR value in patients with diabetes and non-diabetes (*P* for log-rank test < 0.001). Moreover, multivariable Cox models demonstrated that the risk of mortality was considerably higher in the lowest quartile of the LMR and no linear trend was observed (*P* > 0.05). Furthermore, the RCS analysis indicated a non-linear decline in the risk of death as LMR values increased (*P* for nonlinearity < 0.001).

**Conclusions:**

Increased LMR is independently related with reduced all-cause mortality in patients with obese hypertension, regardless of whether they have combined diabetes.

## Introduction

Metabolic syndrome is an increasingly common condition that includes obesity, dyslipidemia, insulin resistance and hypertension (1). The prevalence and incidence of obesity is on the rise and poses a significant population health burden worldwide (2). More than two thirds of deaths were linked to obesity (3).

Hypertension and diabetes mellitus (DM) are considered to be serious public health problems that have a significant negative impact on human life and increase health expenditure (4–6). Obesity, hypertension, and DM are major risk factors for cardiovascular disease and all-cause mortality (7–9). Therefore, it is important to identify relevant risk factor to avoid, delay or reduce deaths related to these diseases.

The ideal predictor should not only have good predictive value, be easy to identify during the diagnostic process, but also be low cost in clinical practice. The lymphocyte/monocyte ratio (LMR) is an easily measured parameter of systemic inflammatory burden and cellular immune response that has been studied as a factor associated with disease severity and prognosis in several clinical conditions (10, 11). Metabolic syndrome is a chronic, low-grade inflammatory condition (12). In patients with diabetes, intermediate products such as advanced glycosylation end products and immune complexes stimulate monocyte infiltration and aggravate cell damage, thus accelerating disease deterioration (13, 14). Monocytes are an important part of the innate immune system and play an active role in endogenous inflammation. It is able to migrate from the bloodstream to different tissues and differentiate into various types, including inflammatory dendritic cells, macrophages, and foam cells. This process triggers the secretion of pro-inflammatory cytokines, the production of matrix metalloproteinases and the formation of reactive oxidizing substances. Therefore, the accumulation of a large number of inflammatory cells with infiltrating ability promotes chronic inflammatory response in the body, leading to endothelial cell dysfunction, degradation and destruction of fibrin cytoskeleton, insulin resistance and so on. This eventually leads to metabolic disorders such as high blood pressure, diabetes and obesity. It had been found that the lymphocyte count was relatively low and the monocyte count was high in patients with cardiovascular disease, which had predictive and prognostic value in myocardial infarction (15, 16). Moreover, there are published studies that have found that reduced LMR is a risk factor for cardiovascular disease (17). In addition, the LMR was an independent predictor of re-hospitalization and long-term major cardiovascular and cerebrovascular adverse events in patients with myocardial infarction with elevated ST-segment after primary percutaneous coronary intervention (18). However, the association between LMR and the risk of all-cause death is unclear in patients of obese hypertension with diabetes or non-diabetes.

Therefore, we conducted this study to try to investigate the association between LMR and all-cause mortality in obese hypertensive patients.

## Methods

### Study population

**Figure 1:**
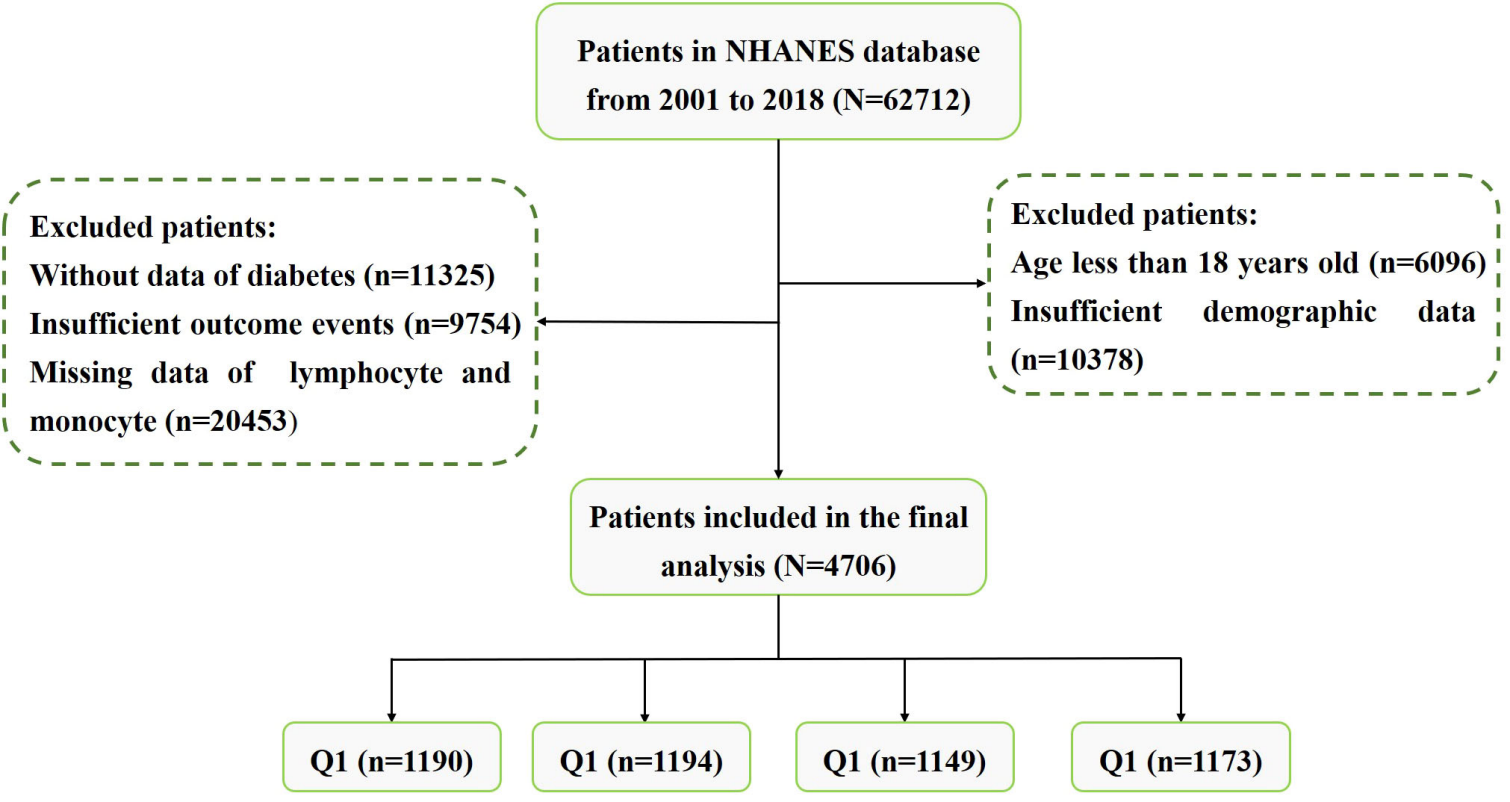
The screening flowchart of participants in our study.

The National Health and Nutrition Examination Survey (NHANES) is a large cross-sectional research program that aims to assess the health and nutritional status of residents in the United States (USA). The program is performed by the Centers for Disease Control (CDC) and Prevention of the USA. The data of our study was obtained from the NHANES official website. We downloaded data from nine cycles of NHANES (2001-2018). In order to protect the rights of participants, NHANES has obtained the informed written consent of all participants. The exclusion criteria were as follows: (1) Patients without lymphocyte and monocyte data. (2) Incomplete information on waist circumference (WC), height, or weight. (3) Lacking of diabetes, fasting plasma glucose (FPG) or glycated hemoglobin (HbA1c) data. (4) Patients aged < 18. (5) Patients without follow-up data. The flowchart for patients screening was presented in **Figure 1**.

### Data collection and definitions

The following covariates are collected: (1) Demographics, including age, gender, race, smoking status (Current smokers: current smokers and have smoked at least 100 cigarettes; Former smoker: has smoked at least 100 cigarettes but does not currently smoke; Never smoked: less than 100 cigarettes), drinking, and education; (2) Laboratory indicators, such as alanine aminotransferase (ALT), aspartate aminotransferase (AST), creatinine, estimated glomerular filtration rate (eGFR), total cholesterol (TC), triglycerides (TG), low density lipoprotein cholesterol (LDL-C), serum uric acid (SUA), FPG, HbA1c, platelet count, neutrophil count, lymphocyte count, monocyte count, and high-sensitivity C-reactive protein (hs-CRP); (3) Measurement indexes, including WC, height, weight, body mass index (BMI), systolic blood pressure (SBP) and diastolic blood pressure (DBP); (4) comorbidities, including heart failure, DM, stroke, coronary artery disease, and hypertension; (5) Endpoint, including follow-up survival status and duration.

Hypertension can be diagnosed if one of the following conditions was met: (1) SBP ≥ 140 mmHg and/or DBP ≥ 90 mmHg, (2) taking antihypertensive medications. The diagnostic criteria for obesity were: (1) BMI ≥ 30 kg/m2, or (2) WC ≥ 85.0 cm for females and ≥ 90.0 cm for males. Obesity-hypertension referred to the presence of both hypertension and obesity. Diabetes was diagnosed if one of the following conditions was met: (1) FPG ≥ 7.0 mmol/L or 2-h post-meal blood glucose level of ≥ 11.1 mmol/L; (2) random blood glucose ≥ 11.1 mmol/L; (3) HbA1c ≥ 6.5%; (4) taking hypoglycemic drugs or using subcutaneous insulin injections; and (5) had been told by a doctor that he had diabetes. The LMR was calculated as lymphocyte count/monocyte count.

### Outcomes

The main endpoint of this study was all-cause mortality during follow-up.

### Statistical analysis

We divided the subjects into four groups (Q1-Q4) based on the quartile of the LMR. Continuous variables were expressed in terms of mean and standard deviation, while categorical variables were presented in terms of frequency and percentage. Baseline characteristics were compared between different groups using univariate ANOVA for continuous variables and Pearson Chi-square test, corrected Chi-square test or Fisher exact test for categorical variables. All-cause mortality was calculated for each LMR quartile array throughout the follow-up period. Kaplan-Meier survival analysis was performed to assess the incidence rate of all-cause death between groups. Log-rank tests were used to evaluate the differences we observed. To evaluate the independent predictive value of the LMR, we developed three multivariate Cox proportional risk models to control for confounding factors. Crude Model was unadjusted, Adjust I Model was adjusted for age, gender, and race, and Adjust II Model was adjusted for gender, age, race, smoking, education, WC, BMI, SBP, DBP, AST, AST, serum creatinine, eGFR, FPG, HbA1c, TC, TG, LDL-C, SUA, hs-CRP, heart failure, stroke and coronary artery disease. The restricted cubic splines (RCS) analysis was employed to further investigate the dose-effect relationship with the LMR and the risk of all-cause mortality in patients of obese hypertension. The receiver operator characteristic curve (ROC) analysis was used to assess the accuracy of the LMR in predicting survival outcomes. A two-tailed p < 0.05 indicated statistical significance.

All analyses were conducted using SPSS statistical software (version 26.0) and R software (version 4.3.2). Moreover, GraphPad Prism software (version 8.0) was employed to make graphs.

## Results

### Baseline characteristics

We eventually included 4706 subjects with obese hypertension by inclusion and exclusion criteria. The baseline characteristics of the study subjects were shown in **Table 1**, stratified by the LMR quartile. The average age of the participants was 59.71 years old and 48.78% were male. Average LMR in the enrolled patients was 3.89 ± 1.97. According to the quartiles of the LMR, the laboratory characteristics at baseline were shown in **Table 2**. Participants with a higher LMR were more likely to be younger, female, former smoker, non-Hispanic Black and lower education, compared with participants in the first quartile. Moreover, significant differences in laboratory indicators were observed between the groups, with participants in the highest quartile showing significantly higher levels of TC, TG, LDL-C, HbA1c and platelet, lower levels serum creatinine, eGFR, SUA, neutrophil and hs-CRP compared with those in the first quartile. In terms of comorbidities, participants in the higher quartile had lower prevalence rates of diabetes, heart failure, coronary artery disease and stroke compared with those in the first quartile. In addition, all-cause mortality decreased gradually (32.61% vs 21.44% vs 14.19% vs 13.04%, *P* < 0.001) with increasing LMR.

**Table 1:** Baseline characteristics according to the LMR quartiles.

Variable	Q1 (n = 1190)	Q2 (n = 1194)	Q3 (n = 1149)	Q4 (n = 1173)	*P* value
Age (years)	65.26 ± 14.06	60.89 ± 14.42	57.84 ± 14.47	54.74 ± 14.59	< 0.001
Male, n (%)	755 (63.44)	629 (52.68)	519 (45.17)	392 (33.42)	< 0.001
Race, n (%)					< 0.001
Mexican American	98 (8.23)	149 (12.48)	180 (15.67)	190 (16.20)	
Other Hispanic	71 (5.96)	85 (7.12)	100 (8.70)	98 (8.35)	
Non-Hispanic White	726 (61.01)	604 (50.59)	487 (42.38)	351 (29.92)	
Non-Hispanic Black	212 (17.82)	267 (22.36)	292 (25.41)	416 (35.46)	
Other Race	83 (6.97)	89 (7.45)	90 (7.83)	118 (10.06)	
Smoking, n (%)					< 0.001
Never smoker	215 (18.07)	228 (19.10)	255 (22.19)	271 (23.10)	
Former smoker	654 (54.96)	589 (49.33)	520 (45.25)	492 (41.94)	
Current smoker	321 (26.97)	377 (31.57)	374 (32.55)	410 (34.95)	
Drinking, n (%)	295 (24.79)	264 (22.11)	255 (22.19)	265 (22.59)	0.775
Education, n (%)					0.002
Less than high school	267 (22.44)	329 (27.47)	326 (28.37)	354 (30.18)	
High school or equivalent	307 (25.80)	289 (24.20)	286 (24.89)	263 (22.42)	
College or above	616 (51.76)	576 (48.24)	537 (46.73)	556 (47.40)	
Anthropometric indicators					
WC (cm)	107.74 ± 14.61	107.42 ± 14.05	106.98 ± 13.80	107.56 ± 14.11	0.618
BMI (kg/m^2^)	31.07 ± 7.06	31.55 ± 6.53	32.05 ± 6.65	32.86 ± 7.32	< 0.001
SBP (mmHg)	134.28 ± 21.11	134.87 ± 22.03	132.96 ± 18.97	133.39 ± 19.47	0.108
DBP (mmHg)	68.21 ± 16.04	71.03 ± 15.42	72.42 ± 14.07	72.77 ± 14.79	< 0.001
Comorbidities					
DM, n (%)	346 (29.08)	307 (25.71)	276 (24.02)	324 (27.62)	0.033
HF, n (%)	156 (13.11)	93 (7.79)	42 (3.66)	58 (4.94)	< 0.001
CAD, n (%)	168 (14.12)	135 (11.31)	69 (6.00)	53 (4.52)	< 0.001
Stroke, n (%)	117 (9.83)	84 (7.04)	62 (5.40)	74 (6.31)	< 0.001
Outcomes					
All-cause mortality, n (%)	388 (32.61)	256 (21.44)	163 (14.19)	153 (13.04)	< 0.001

WC, waist circumference; BMI, body mass index; SBP, systolic blood pressure; DBP, diastolic blood pressure; DM, diabetes mellitus; HF, heart failure; CAD, coronary artery disease

**Table 2:** Baseline levels of laboratory indicators according to the LMR quartiles.

Laboratory parameters	Q1	Q2	Q3	Q4	*P* value
	(n = 1190)	(n = 1194)	(n = 1149)	(n = 1173)	
ALT (U/L)	24.95 ± 18.55	24.89 ± 16.43	25.42 ± 14.15	24.74 ± 14.13	0.764
AST (U/L)	26.51 ± 16.81	25.74 ± 16.22	25.10 ± 11.08	24.57 ± 11.73	0.063
Scr (mg/dL)	1.14 ± 1.02	1.01 ± 0.55	0.93 ± 0.55	0.88 ± 0.35	< 0.001
eGFR (ml/min/1.73m^2^)	72.11 ± 23.76	78.02 ± 22.84	83.28 ± 21.57	86.07 ± 21.51	< 0.001
TC (mmol/L)	4.74 ± 1.13	4.95 ± 1.10	5.03 ± 1.05	5.03 ± 1.12	< 0.001
TG (mmol/L)	1.52 ± 1.00	1.64 ± 1.30	1.68 ± 1.31	1.69 ± 1.28	0.002
LDL-C (mmol/L)	2.71 ± 0.96	2.87 ± 0.92	2.94 ± 0.88	3.06 ± 0.95	< 0.001
HDL-C (mmol/L)	1.34 ± 0.44	1.33 ± 0.40	1.33 ± 0.40	1.34 ± 0.36	0.964
FPG (mmol/L)	6.62 ± 2.61	6.26 ± 2.18	6.58 ± 2.75	6.51 ± 2.97	0.315
HbA1c (%)	6.05 ± 1.11	6.03 ± 1.22	6.10 ± 1.35	6.21 ± 1.42	< 0.001
SUA (mg/dl)	6.24 ± 1.55	5.89 ± 1.54	5.83 ± 1.45	5.73 ± 1.41	< 0.001
PLT (1000 cells/uL)	230.79 ± 70.89	238.63 ± 65.01	246.85 ± 69.11	255.19 ± 74.66	< 0.001
Neu (1000 cells/uL)	4.75 ± 1.96	4.31 ± 1.61	4.01 ± 1.52	3.75 ± 1.48	< 0.001
Lym (1000 cells/uL)	1.48 ± 0.47	1.87 ± 0.45	2.18 ± 0.35	2.72 ± 0.63	< 0.001
Mono (1000 cells/uL)	0.70 ± 0.25	0.58 ± 0.14	0.53 ± 0.31	0.43 ± 0.18	< 0.001
LMR	2.14 ± 0.46	3.19 ± 0.25	4.09 ± 0.28	6.16 ± 0.54	< 0.001
hs-CRP (mg/L)	6.72 ± 1.92	5.70 ± 1.02	4.75 ± 1.41	4.28 ± 1.44	0.007

ALT, alanine aminotransferase; AST, aspartate aminotransferase; Scr, serum creatinine; eGFR, estimated glomerular filtration rate; TG, triglyceride; TC, total cholesterol; LDL-C, low density lipoprotein cholesterol; HDL-C, high density lipoprotein cholesterol; FPG, fasting plasma glucose; HbA1c, glycosylated hemoglobin; SUA, serum uric acid; PLT, platelet; Neu, neutrophile; Lym, lymphocyte; Mono, monocyte; LMR, lymphocyte to monocyte ratio; hs-CRP, high-sensitivity C-reactive protein

### Correlation between the LMR and all−cause mortality

**Figure 2:**
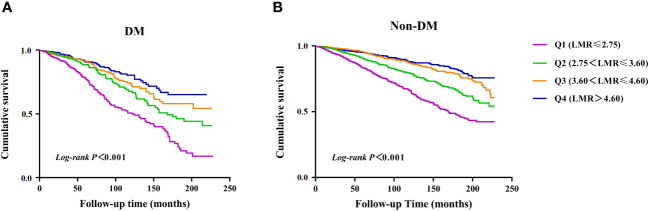
Kaplan–Meier survival analysis curves for all-cause mortality. **(A)** diabetes, **(B)** non- diabetes.

There were 960 incident cases of all-cause mortality during follow-up (median follow-up of 90 months). The Kaplan-Meier survival analysis curves showed the prevalence of all-cause mortality in several groups that have been divided based on the LMR quartiles in **Figure 2**. Participants with a higher LMR demonstrated a significantly higher survival rate compared to those with a lower LMR in DM (*P* for log-rank test < 0.001) (**Figure 2A**). Similarly, there was significant difference observed in patients with non-DM (*P* for log-rank test < 0.001) (**Figure 2B**).

Further, Cox analysis revealed a significant relationship between the LMR and all-cause mortality in the crude model [HR (95% CI): 0.78 (0.72-0.86), P < 0.001] and the adjusted models [Adjusted I: HR (95% CI): 0.89 (0.82-0.98), P = 0.006; Adjusted II: HR (95% CI): 0.88 (0.80-0.98), P = 0.017] in patients with DM when the LMR was considered a continuous variable. However, there was a significant association between LMR and all-cause mortality only in the crude model [HR (95% CI): 0.73 (0.68-0.77), P < 0.001] in patients without DM (**Table 3**). When LMR was treated as a categorical variable, it was observed that, compared with patients in the Q1 group, patients in the highest LMR group (Q4) with DM suggested a significantly decreased risk of all-cause mortality in three established Cox models, as showed by the following findings: crude model [HR (95% CI): 0.46 (0.26-0.49), P < 0.001], Adjust I model [HR (95% CI): 0.61 (0.43-0.85), P = 0.004], and Adjust II model [HR (95% CI): 0.59 (0.39-0.86), P = 0.007]. However, there was non-trend decreasing risk of mortality with elevated the LMR, as shown by the results of the trend test (P for trend > 0.05) (**Table 3**). Similar results were observed in patients without DM [crude model: HR (95% CI): 0.31 (0.24-0.39), P < 0.001; Adjust I model: HR (95% CI): 0.74 (0.57-0.96), P = 0.021; Adjust II model: HR (95% CI): 0.71 (0.54-0.93), P = 0.014; P for trend > 0.05] (**Table 3**).

**Table 3:** Association between quartiles of LMR with risk of all-cause mortality.

LMR	Crude	*P* value	Adjust I	*P* value	Adjust II	*P* value
	HR (95% CI)		HR (95% CI)		HR (95% CI)	
DM						
Per 1 Unit increase	0.78 (0.72-0.86)	< 0.001	0.89 (0.82-0.98)	0.006	0.88 (0.80-0.98)	0.017
Quartiles						
Q1	Ref		Ref		Ref	
Q2	0.53 (0.40-0.71)	< 0.001	0.69 (0.51-0.94)	0.018	0.64 (0.46-0.92)	0.014
Q3	0.41 (0.30-0.56)	< 0.001	0.60 (0.43-0.84)	0.003	0.65 (0.44-0.92)	0.017
Q4	0.46 (0.26-0.49)	< 0.001	0.61 (0.43-0.85)	0.004	0.59 (0.39-0.86)	0.007
*P* for trend		0.428		0.621		0.414
**Non-DM**						
Per 1 Unit increase	0.73 (0.68-0.77)	< 0.001	0.94 (0.88-1.00)	0.060	0.95 (0.89-1.01)	0.090
Quartiles						
Q1	Ref		Ref		Ref	
Q2	0.58 (0.48-0.69)	< 0.001	0.80 (0.65-0.97)	0.022	0.81 (0.65-0.98)	0.038
Q3	0.34 (0.27-0.42)	< 0.001	0.58 (0.46-0.73)	< 0.001	0.63 (0.48-0.80)	< 0.001
Q4	0.31 (0.24-0.39)	< 0.001	0.74 (0.57-0.96)	0.021	0.71 (0.54-0.93)	0.014
*P* for trend		0.053		0.313		0.086

CI, confidence interval; HR, hazard ratio; LMR, Lymphocyte to monocyte ratio; DM, diabetes mellitus.

Crude, Unadjusted model.

Adjust I: adjusted for gender, age, race.

Adjust II: adjusted for gender, age, race, smoking, education, WC, BMI, SBP, DBP, AST, AST, serum creatinine, eGFR, FPG, HbA1c, TC, TG, LDL-C, SUA, neutrophil count, platelet count, hs-CRP, heart failure, stroke and coronary artery disease.

### The detection of nonlinear relationship

**Figure 3:**
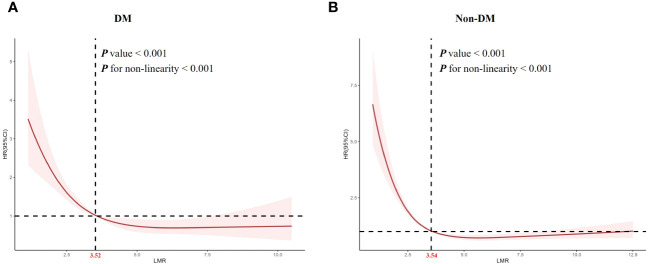
Restricted cubic spline analysis of LMR with all-cause mortality. **(A)** diabetes, **(B)** non- diabetes.

Considering that multivariate Cox proportional hazard analysis suggested a non-linear relationship between the baseline LMR and all-cause mortality, we used RCS analysis to further explore this association in patients with obesity-related hypertension (**Figure 3**). We studied the population of diabetes and non-diabetes separately. The RCS analysis revealed a non-linear association between LMR and all-cause mortality in both diabetic (P for non-linearity < 0.001, **Figure 3A**) and non-diabetic patients (P for non-linearity < 0.001, **Figure 3B**).

### Subgroup analysis

**Figure 4:**
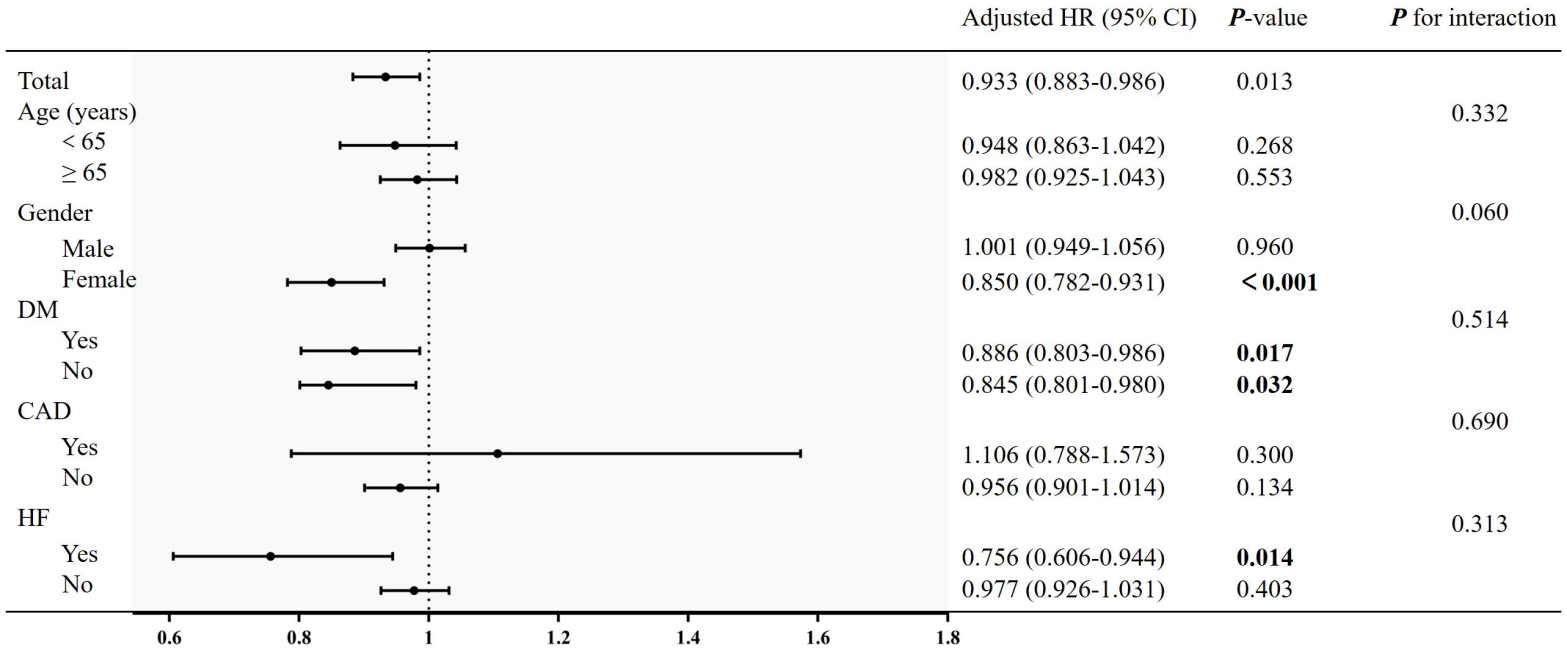
Subgroup analysis of the association between LMR and all-cause mortality. Adjusted for age, gender, race, smoking, education, WC, BMI, SBP, DBP, AST, AST, serum creatinine, eGFR, FPG, HbA1c, TC, TG, LDL-C, SUA, neutrophil count, platelet count, hs-CRP, heart failure, stroke and coronary artery disease.

To evaluate the specific effect of the LMR on the outcomes, stratification was performed according to age, gender, DM, heart failure and coronary artery disease in **Figure 4**. Although female was a protective factor for all-cause mortality, there was no interaction between the gender [female: HR (95% CI): 0.850 (0.782-0.931), *P* < 0.001; *P* for interaction > 0.05). Similarly, there was no significant interaction in diabetes subgroups and other subgroups (*P* for interaction > 0.05).

### ROC curve analysis of LMR

**Figure 5:**
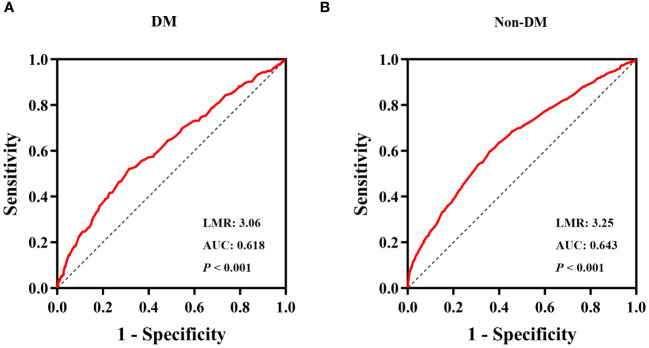
ROC Curve analysis for LMR predicted all-cause mortality. **(A)** diabetes, **(B)** non- diabetes.

The ROC curve for the LMR in predicting all-cause mortality was depicted in **Figure 5**. The ROC curve revealed a moderate ability of LMR to predict mortality in obesity-related hypertension with DM [AUC=0.618, 95% CI (0.582-0.655), *P* < 0.001, **Figure 5A**] and without DM [AUC=0.643, 95% CI (0.625-0.674), *P* < 0.001, **Figure 5B**]. The cut-off values were 3.06 and 3.25, respectively. The sensitivity was 0.686 and 0.643, while the specificity was 0.522 and 0.595, respectively.

## Discussion

The study was conducted to explore the association between the LMR and survival outcomes in patients with obese hypertension. In our study, among the 4706 participants from nine NHANES cycles (2001-2018), a decreased LMR was strongly related to all-cause mortality and was an independent risk factor for reduced survival. Moreover, there was a non-linear relationship between LMR and all-cause mortality in both diabetic and non-diabetic patients.

Since LMR consists of lymphocyte and monocyte counts, it has the advantage of being relatively inexpensive and can be obtained routinely. A low lymphocyte count indicates a persistent, relatively deficient immune state, and a high monocyte count indicates a non-specific or systemic inflammatory state (19–22). As a composite parameter reflecting two opposing immune and inflammatory pathways, LMR is more predictive than lymphocyte or monocyte alone. Inflammation plays an important role in the pathophysiology of many metabolic diseases (23). Moreover, previous studies demonstrated that activation of the immune system and chronic inflammation were involved in the occurrence and development of these diseases (24). LMR has been reported to be associated not only with an increased risk of death from multiple malignant diseases, but also with a poor prognosis for coronary artery disease (25–27).. In our study, compared with participants of the higher LMR, the lower LMR had higher levels of neutrophil and hs-CRP and a higher prevalence of metabolic disorders such as diabetes, heart failure and coronary artery disease. This suggested that lower LMR may be associated with higher levels of inflammation and adverse outcomes.

Our findings of association between the LMR and all-cause mortality in the obese hypertension patients were somewhat consistent with recent studies (28–31). Previous study has demonstrated that the lower LMR was associated with cardiovascular mortality and all-cause mortality in adult asthma patients (28). In another study, the researchers found that patients with pulmonary embolism had a significantly increased risk of 30-day all-cause mortality with the reduction of LMR (29). Furthermore, poor overall survival was strongly associated with low LMR in patients with thyroid cancer (30). A study on the prognosis of patients with heart failure also showed that a lower LMR significantly increased the risk of all-cause death within 6 months (31). Notably, they did not further explore the specific correlation between LMR and mortality, whereas our study demonstrated that a non-linear relationship between the LMR and all-cause mortality. The possible mechanism of the above findings is: proinflammatory cytokines activate lymphocytes and monocytes (32). This activated cell is a potential source of pro-inflammatory cytokines, leading to further activation of these cells, which contributes to systemic inflammation in obese hypertensive patients. High levels of proinflammatory cytokines may cause some adverse effects in patients, including myocardial remodeling and promoting arrhythmia (33). Ultimately, there was an increased risk of adverse outcome events.

To our knowledge, this was the first study that explored the prognostic effect of LMR on patients with obese hypertension. However, there are some limitations in this study. Firstly, this was a single-center retrospective study, so potential bias could be present. Moreover, the sample size was not particularly large, and the incidence of death was relatively low. Future large-sample and prospective studies are warranted to strengthen our findings. Finally, there were no other indicators of inflammation levels or immune status except hs-CRP. Thus, we cannot reveal the exact pathophysiological mechanisms underlying LMR. Therefore, basic experiments or animal experiments are needed to further explore the specific mechanism.

## Conclusions

We found that LMR is a valuable tool for predicting the risk of all-cause mortality in patients with obese hypertension combined with diabetes or without diabetes, and that the relationship between LMR and mortality is non-linear. Thus, LMR may be helpful in predicting risk and assessing prognosis in these patients. In addition, more research is needed to explore whether intervening with LMR can contribute to improve clinical outcomes for these patients.

## Data availability statement

The original contributions presented in the study are included in the article/supplementary material. Further inquiries can be directed to the corresponding authors.

## Author contributions

LW: Writing – original draft, Writing – review & editing. JG: Investigation, Writing – original draft. BL: Writing – original draft. YF: Writing – original draft. ZY: Writing – original draft. SG: Writing – original draft. ZS: Writing – original draft. ZZ: Writing – original draft. JH: Writing – original draft. CW: Writing – original draft. WM: Writing – review & editing. FW: Writing – review & editing.

## References

[1] Ricci G Pirillo I Tomassoni D Sirignano A Grappasonni I. Metabolic syndrome, hypertension, and nervous system injury: Epidemiological correlates. Clin Exp Hypertens. (2017) 39:8–16. doi: 10.1080/10641963.2016.1210629 28071980

[2] Roberto CA Swinburn B Hawkes C Huang TT Costa SA Ashe M. Patchy progress on obesity prevention: emerging examples, entrenched barriers, and new thinking. Lancet. (2015) 385:2400–9. doi: 10.1016/S0140-6736(14)61744-X 25703111

[3] Afshin A Forouzanfar MH Reitsma MB Sur P Estep K Lee A. Health effects of overweight and obesity in 195 countries over 25 years. N Engl J Med. (2017) 377:13–27. doi: 10.1056/NEJMoa1614362 28604169 PMC5477817

[4] Mills KT Stefanescu A He J. The global epidemiology of hypertension. Nat Rev Nephrol. (2020) 16:223–37. doi: 10.1038/s41581-019-0244-2 PMC7998524 32024986

[5] Kehlenbrink S Smith J Ansbro É Fuhr DC Cheung A Ratnayake R. The burden of diabetes and use of diabetes care in humanitarian crises in low-income and middle-income countries. Lancet Diabetes Endocrinol. (2019) 7:638–47. doi: 10.1016/S2213-8587(19)30082-8 30878268

[6] Mancia G Cappuccio FP Burnier M Coca A Persu A Borghi C. Perspectives on improving blood pressure control to reduce the clinical and economic burden of hypertension. J Intern Med. (2023) 294:251–68. doi: 10.1111/joim.13678 37401044

[7] Wu Y Zhang H Jiang D Yin F Guo P Zhang X. Body mass index and the risk of abdominal aortic aneurysm presence and post-operative mortality: a systematic review and dose-response meta-analysis. Int J Surg. (2024). doi: 10.1097/JS9.0000000000001125 PMC11020033 38320094

[8] Hardy ST Loehr LR Butler KR Chakladar S Chang PP Folsom AR. Reducing the blood pressure-related burden of cardiovascular disease: impact of achievable improvements in blood pressure prevention and control. J Am Heart Assoc. (2015) 4:e002276. doi: 10.1161/JAHA.115.002276 26508742 PMC4845128

[9] Ettehad D Emdin CA Kiran A Anderson SG Callender T Emberson J. Blood pressure lowering for prevention of cardiovascular disease and death: a systematic review and meta-analysis. Lancet. (2016) 387:957–67. doi: 10.1016/S0140-6736(15)01225-8 26724178

[10] Nøst TH Alcala K Urbarova I Byrne KS Guida F Sandanger TM Johansson M. Systemic Inflammation Markers Cancer incidence UK Biobank. Eur J Epidemiol. (2021) 36:841–8. doi: 10.1007/s10654-021-00752-6 PMC8416852 34036468

[11] Karauzum I Karauzum K Acar B Hanci K Bildirici HIU Kilic T Ural E. Predictive value of lymphocyte-to-monocyte ratio in patients with contrast-induced nephropathy after percutaneous coronary intervention for acute coronary syndrome. J Transl Int Med. (2021) 9:123–30. doi: 10.2478/jtim-2021-0024 PMC8386327 34497751

[12] Ye D Miyoshi A Ushitani T Kadoya M Igeta M Konishi K. RAGE in circulating immune cells is fundamental for hippocampal inflammation and cognitive decline in a mouse model of latent chronic inflammation. Brain Behav Immun. (2024) 116:329–48. doi: 10.1016/j.bbi.2023.12.022 38142917

[13] Lim AK Tesch GH. Inflammation in diabetic nephropathy. Mediators Inflammation. (2012) 2012:146154. doi: 10.1155/2012/146154 PMC3432398 22969168

[14] Kocak MZ Aktas G Duman TT Atak BM Kurtkulagi O Tekce H. Monocyte lymphocyte ratio As a predictor of Diabetic Kidney Injury in type 2 Diabetes mellitus; The MADKID Study. J Diabetes Metab Disord. (2020) 19:997–1002. doi: 10.1007/s40200-020-00595-0 33553019 PMC7843868

[15] van der Laan AM Hirsch A Robbers LF Nijveldt R Lommerse I Delewi R. A proinflammatory monocyte response is associated with myocardial injury and impaired functional outcome in patients with ST-segment elevation myocardial infarction: monocytes and myocardial infarction. Am Heart J. (2012) 163:57–65. doi: 10.1016/j.ahj.2011.09.002 22172437

[16] Núñez J Núñez E Bodí V Sanchis J Mainar L Miñana G. Low lymphocyte count in acute phase of ST-segment elevation myocardial infarction predicts long-term recurrent myocardial infarction. Coron Artery Dis. (2010) 21:1–7. doi: 10.1097/MCA.0b013e328332ee15 20050312

[17] Murat SN Yarlioglues M Celik IE Kurtul A Duran M Kilic A Oksuz F. The relationship between lymphocyte-to-monocyte ratio and bare-metal stent in-stent restenosis in patients with stable coronary artery disease. Clin Appl Thromb Hemost. (2017) 23:235–40. doi: 10.1177/1076029615627340 26759373

[18] Wang Q Ma J Jiang Z Wu F Ping J Ming L. Association of lymphocyte-to-monocyte ratio with in-hospital and long-term major adverse cardiac and cerebrovascular events in patients with ST-elevated myocardial infarction. Med (Baltimore). (2017) 96:e7897. doi: 10.1097/MD.0000000000007897 PMC5572030 28834908

[19] Shen S Zhang M Wang X Liu Q Su H Sun B. Single-cell RNA sequencing reveals S100a9(hi) macrophages promote the transition from acute inflammation to fibrotic remodeling after myocardial ischemia−reperfusion. Theranostics. (2024) 14:1241–59. doi: 10.7150/thno.91180 PMC10845204 38323308

[20] Avolio F Martinotti S Khavinson VK Esposito JE Giambuzzi G Marino A. Peptides regulating proliferative activity and inflammatory pathways in the monocyte/macrophage THP-1 cell line. Int J Mol Sci. (2022) 23:3607. doi: 10.3390/ijms23073607 35408963 PMC8999041

[21] Ryu H Bi TM Pulliam TH Sarkar K Church CD Kumar N. Merkel cell polyomavirus-specific and CD39(+)CLA(+) CD8 T cells as blood-based predictive biomarkers for PD-1 blockade in Merkel cell carcinoma. Cell Rep Med. (2024) 5:101390. doi: 10.1016/j.xcrm.2023.101390 38340724 PMC10897544

[22] Abadie K Clark EC Valanparambil RM Ukogu O Yang W Daza RM. Reversible, tunable epigenetic silencing of TCF1 generates flexibility in the T cell memory decision. Immunity. (2024) 57:271–86. doi: 10.1016/j.immuni.2023.12.006 PMC10922671 38301652

[23] Iadecola C Anrather J. The immunology of stroke: from mechanisms to translation. Nat Med. (2011) 17:796–808. doi: 10.1038/nm.2399 21738161 PMC3137275

[24] Esser N Legrand-Poels S Piette J Scheen AJ Paquot N. Inflammation as a link between obesity, metabolic syndrome and type 2 diabetes. Diabetes Res Clin Pract. (2014) 105:141–50. doi: 10.1016/j.diabres.2014.04.006 24798950

[25] Kiris T Çelik A Variş E Akan E Akyildiz ZI Karaca M. Association of lymphocyte-to-monocyte ratio with the mortality in patients with ST-elevation myocardial infarction who underwent primary percutaneous coronary intervention. Angiology. (2017) 68:707–15. doi: 10.1177/0003319716685480 28056530

[26] Ji H Li Y Fan Z Zuo B Jian X Li L Liu T. Monocyte/lymphocyte ratio predicts the severity of coronary artery disease: a syntax score assessment. BMC Cardiovasc Disord. (2017) 17:90. doi: 10.1186/s12872-017-0507-4 28359298 PMC5374608

[27] Yang Y Liang Y Sadeghi F Feychting M Hamar N Fang F. Risk of head and neck cancer in relation to blood inflammatory biomarkers in the Swedish AMORIS cohort. Front Immunol. (2023) 14:1265406. doi: 10.3389/fimmu.2023.1265406 37876941 PMC10590876

[28] Zhu N Lin S Yu H Liu F Huang W Cao C. Naples prognostic score as a novel prognostic prediction indicator in adult asthma patients: A population-based study. World Allergy Organ J. (2023) 16:100825. doi: 10.1016/j.waojou.2023.100825 37954399 PMC10632111

[29] Zhu N Lin S Cao C. A novel prognostic prediction indicator in patients with acute pulmonary embolism: Naples prognostic score. Thromb J. (2023) 21:114. doi: 10.1186/s12959-023-00554-8 37932805 PMC10629175

[30] Ahn J Song E Oh HS Song DE Kim WG Kim TY. Low lymphocyte-to-monocyte ratios are associated with poor overall survival in anaplastic thyroid carcinoma patients. Thyroid. (2019) 29:824–9. doi: 10.1089/thy.2018.0684 30864902

[31] Silva N Bettencourt P Guimarães JT. The lymphocyte-to-monocyte ratio: an added value for death prediction in heart failure. Nutr Metab Cardiovasc Dis. (2015) 25:1033–40. doi: 10.1016/j.numecd.2015.07.004 26482565

[32] Tamariz L Hare JM. Inflammatory cytokines in heart failure: roles in aetiology and utility as biomarkers. Eur Heart J. (2010) 31:768–70. doi: 10.1093/eurheartj/ehq014 20172914

[33] Prabhu SD. Cytokine-induced modulation of cardiac function. Circ Res. (2004) 95:1140–53. doi: 10.1161/01.RES.0000150734.79804.92 15591236

